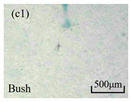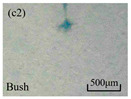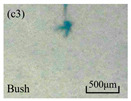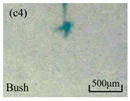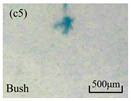# Effect of sPP Content on Electrical Tree Growth Characteristics in PP-Blended Cable Insulation

**DOI:** 10.3390/ma13235360

**Published:** 2020-11-26

**Authors:** Shuofan Zhou, Fan Yu, Wei Yang, Zhonglei Li, Zhaoliang Xing, Mingsheng Fan, Tao Han, Boxue Du

**Affiliations:** 1 State Key Laboratory of Advanced Power Transmission Technology, Global Energy Interconnection Research Institute Co. Ltd., Beijing 102209, China; nmxxzsf@tju.edu.cn (S.Z.); fanyu@163.com (F.Y.); weiyang@163.com (W.Y.); zhlx@163.com (Z.X.); 2 School of Electrical and Information Engineering, Tianjin University, Tianjin 300072, China; fanmingsheng@tju.edu.cn (M.F.); hant@tju.edu.cn (T.H.)

**Keywords:** cable insulation, polypropylene, blend, electrical tree, breakdown strength, crystallization

## Abstract

This paper aims at investigating the electrical tree characteristics of isotactic polypropylene (iPP)/syndiotactic polypropylene (sPP) blends for thermoplastic cable insulation. PP blended samples with sPP contents of 0, 5, 15, 30, and 45 wt% are prepared, and electrical treeing experiments are implemented under alternating current (AC) voltage at 50, 70, and 90 °C. Experimental results show that with the incorporation of sPP increasing to 15 wt%, the inception time of electrical tree increases by 8.2%. The addition of sPP by 15% distinguishes an excellent performance in inhibiting electrical treeing, which benefits from the ability to promote the fractal dimension and lateral growth of branches. Further increase in sPP loading has a negative effect on the electrical treeing resistance of blended insulation. It is proved by DSC and POM that the addition of sPP promotes the heterogeneous crystallization the of PP matrix, resulting in an increasing density of interfacial regions between crystalline regions, which contains charge carrier traps. Charges injected from an electrode into a polymer are captured by deep traps at the interfacial regions, thus inhibiting the propagation of electrical tree. It is concluded that the modification of crystalline morphology by 15 wt% sPP addition has a great advantage in electrical treeing resistance for PP-based cable insulation.

## 1. Introduction

As a promising type of environmentally friendly and recyclable insulation, polypropylene (PP) has attracted wide attention for its advantages of excellent electrical and heat resistance properties [1,2]. Nowadays, PP-based insulation cable has been in use by thousands of kilometers in medium voltage power systems in Europe. Italy and Netherlands are the first countries to promote modified PP material in cable insulation. Spain and Finland have also successively promoted modified PP as cable insulation material in medium voltage power systems [2,3]. Under the continuous action of the electric field, the insulation will gradually deteriorate and may induce breakdown, which may threaten the normal operation of the power system. Inhibiting the initiation and growth of electrical tree in polypropylene insulation is the basis of accelerating the practical application of polypropylene insulation [4].

The degradation mechanism and suppression measures of electrical tree in polypropylene insulation have been analyzed recently. Dynamic growth characteristics of electrical tree in PP under direct current (DC) and impulse electric field indicates that the growth rate and accumulated damage of the electrical tree increased with the increase of the pulse voltage amplitude [5]. The growth rate and accumulated damage increased with the increase of temperature [6]. Grafting, nano modification, and blending have been widely studied as methods to inhibit the insulation failure of PP. The grafting of polypropylene indicates that deep traps induced by grafted group is due to the different electronic band structure between the grafted group and the original polymer. Charge carriers are easier to be trapped by deep traps introduced by the grafted groups, thus inhibiting charge transport [7]. Different nanoparticles (MgO, ZnO, and Al_2_O_3_) on the electrical properties of iPP have been examined. It was found that the dielectric constant of all samples increases with the increase of the mass fraction of nanoparticles. However, the dielectric loss of MgO and ZnO nanocomposites is lower than that of pure PP, and the dielectric loss of Al_2_O_3_ nanocomposites is equivalent to that of pure PP [8,9,10]. The blending method which is easy to accept in industry is a research hotspot to improve the properties of polypropylene. Blending iPP with sPP has confirmed the improvement of the breakdown field strength, which reveals the application possibility of iPP/sPP cable insulation [11]. The morphology and mechanical behavior of iPP/sPP blends are investigated and it is concluded that spherulites of blends convert imperfect accompanied by decreasing of the spherulite size with increasing the addition of sPP [12]. Blending iPP with sPP and atactic polypropylene (aPP) will increase the crystallization degree of the iPP matrix and form more deep traps at the boundary of perfect spherulites, leading to the reduction of mean free path of charges [13,14]. These initial works have clearly confirmed the feasibility of the modification method by bending iPP with sPP in the thermoplastic cable insulation. However, there is no systematic study on the initial probability and growth rate of electrical tree in iPP/sPP blend insulation, which is an essential and indispensable process of promoting its application.

Compatibility is an important factor to determine the properties of blends. It is found that iPP/sPP blends mixed in equal proportion are phase separated [15,16]. A state of mixture borders on phase separation, which indicates that the blend iPP/sPP is immiscible [17]. Further research indicated that there is a dispersion structure in the skin layer and a continuous structure in the core layer. Due to the different crystallization temperature of iPP and sPP, migration in the blends and the inhomogeneity of the components contribute to the aggregation structure. Research confirmed the immiscibility of the blends under various compositions, but no phase separation was observed for components containing a small amount of sPP (10 wt%) [18]. A minor component is dispersed as a nodule in the main component of the blend, which promotes the heterogeneous nucleation [19]. It can be inferred that co-crystallinity of iPP/sPP is possible when the addition amount of sPP is less than a specific value.

The purpose of this article is to improve the electrical tree inhibiting characteristics of iPP insulation by blending sPP. In this paper, the influences of sPP content on the inception and growth of electrical tree and AC breakdown strength are investigated. Differential scanning calorimetry (DSC) is employed to characterize the crystal morphology of blended insulation. The trap energy level characteristics are analyzed by using isothermal discharge current (IDC) experiments. A discussion based on the electric field affected by trapped charge is provided to help in understanding the mechanism of microscopic crystallization on macro degradation.

## 2. Experimental Arrangement

### 2.1. Sample Preparation and Characteristics

In this study, iPP was manufactured by Oasis Petroleum Inc (HP456J, Houston, TX, USA, density: 0.90 g/cm^3^, melting point: 161 °C) while sPP was manufactured by Borealis (BD950, Vienna, Austria, density: 0.90 g/cm^3^, melting point: 142 °C). The iPP/sPP blended samples were prepared with the weight ratio of sPP by 0, 5, 15, 30, and 45 wt%. The catalogue of blend samples investigated in this work is shown in Table 1. Raw materials were dried for 24 h in a drying oven in advance. In order to completely mix the raw materials, iPP and sPP were melted at twin roll mill at 180 °C and fully blended by shear stress for 15 min. The flat plate specimen with the size of 80 mm × 30 mm × 1.2 mm was suppressed by a plate vulcanizing press machine at 160 °C with 15 MPa pressure for 10 min. The sample prepared was heated to the melting state with needle (made of stainless steel) insertion in order to carry out the electrical tree experiment. The protrusion of the tail of the needle contacted the edge of the model, thus ensuring the distance of 2 mm between the needle tip and the bottom. The diameter of the needle body was 300 μm and the radius of curvature of the needle tip was 3 μm. The unqualified samples with air cavities have been removed before our experiment. The bottom surface of the sample was pasted with copper foil for the electrical tree experiment [6].

**Figure 1:**
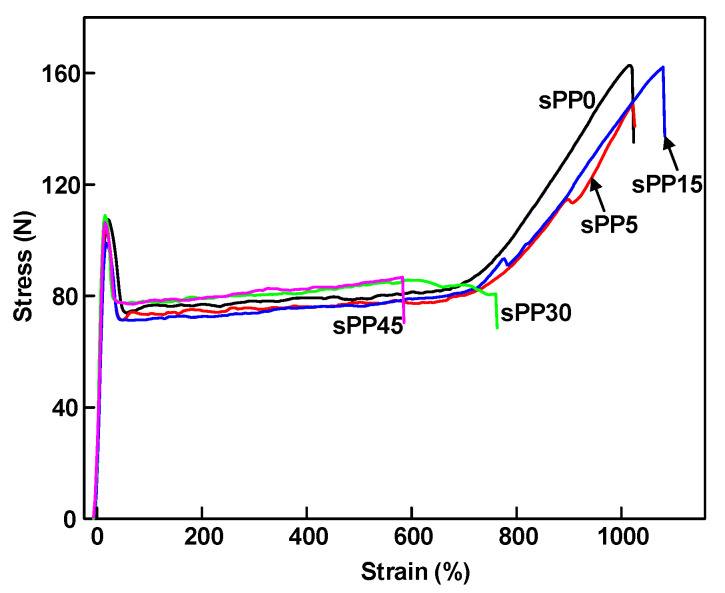
Stress–strain curves of iPP/sPP blends.

Electronic universal material testing machine (WD-D3, Shanghai Zhuoji instrument and Equipment Co., Ltd., Shanghai, China) was used to stretch the dumbbell shaped specimen at room temperature at a tensile rate of 200 mm/min until the specimen is broken. The dumbbell shaped specimen was made by punching machine, with a length of 30 mm, a width of 4 mm, and a thickness of 1 mm. The results of elongation at break and tensile strength of samples are shown in Figure 1. As the content of sPP increased, the elongation at break of iPP/sPP blends increased slightly and then decreased. When the content of sPP reached 15 wt%, the elongation at break and tensile strength reached the maximum. With the further increase of sPP content, the phase separation effect between iPP and sPP made the elongation at break decrease rapidly. Compared with sPP0, the elongation at break of sPP15 was increased by 5.5%, which exhibits better mechanical properties than pure PP for cable insulation.

### 2.2. Electrical Treeing and Breakdown Tests

The electrical tree experiment system was mainly composed of a high voltage alternating current (HVAC) power supply (OYHS-9803, Ouyang Huasi Power Co., Ltd., Shenzhen, China), needle-plate electrode, imaging system (computer, microscope and cold light source) and heating system (insulating oil, temperature sensor (temperature coefficient resistance) and a control unit (XH-W3002, Square Electronic Technology Co., Ltd., Shenzhen, China). The test voltage was 6 kV with a frequency of 50 Hz, and a total harmonic distortion of less than 1%. The temperature of the experiment was set at 50, 70, and 90 °C. The samples to be tested were placed in the drying oven (Suote Hongxiang Co., Ltd., Nanjing, China) and heated sufficiently to maintain the temperature of the experiment. Photos of electrical tree of samples were taken by the charge coupled device (CCD) lens (TD-HU608A, Sanqtid Co., Ltd., Shenzhen, China) and saved in the computer for later analysis. Each sample was tested 5 times to obtain average values.

**Figure 2:**
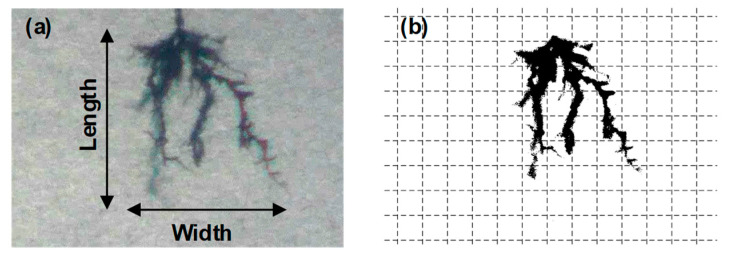
Processing method of electrical tree image. (**a**) The length and width of the electrical tree; (**b**) Image processing method for outlining the treeing area by a binary image.

In this paper, the degradation level of insulation is described by tree length, tree width, expansion coefficient, and fractal dimension (FD). The length and width of the electrical tree are illustrated in Figure 2a. The expansion coefficient is defined as the ratio of the width to the length of the electrical tree. FD is a parameter for quantitative analysis of material deterioration caused by the tree propagation. Figure 2b presents the image processing method for outlining the treeing area by a binary image [20]. The image is divided into a grid of square cells with side length *r*. The minimum number of squares required to overlay the pattern is *N_r_*. Then, the fractal dimension can be calculated by [20]:

(1) $$FD=\underset{r\to 0}{\lim}\frac{\ln(N_{r})}{-\ln(r)}$$

The AC breakdown strength measurements were performed at 50, 70, and 90 °C, by using a pair of ball electrode with a diameter of 20 mm. Samples were immersed in insulating oil to avoid surface flashover. The experiment was carried out 15 times for each group of samples and the Weibull distribution of results was plotted [14].

### 2.3. Trap Level Distribution Characterized by IDC Test

The trap level distributions of iPP/sPP blends were characterized by the IDC method [16]. Samples were polarized under the voltage of 30 kV/mm for 45 min at 323 K, and then depolarized for 40 min to detect the isothermal discharge current (*I*). The current values were recorded by an ammeter (Keithley 6514). The equation below was used to calculate the trap density *N_t_*(*E*) varying with trap level *E_t_*, of which the detailed process had been introduced previously [21,22].

(2) $$\frac{N_{t}(E)}{E_{t}}=\frac{It}{\frac{ekTl^{2}}{2d}f_{0}(E)kT\ln(vt)}$$

where *t* represents the depolarization time; *e* is the electronic charge quantity; *l* is the depth of injected electrons; *k* is the Boltzmann constant; *T* is absolute temperature; *d* represents the sample thickness; *f*_0_(*E*) is equal to 1, indicating the charge traps are fulfilled in this experiment; *v* is the escape frequency of trapped electrons, of which the value is 10^12^ s^−1^ for PP.

## 3. Results

### 3.1. Effect of sPP Content on Electrical Tree Inception

**Figure 3:**
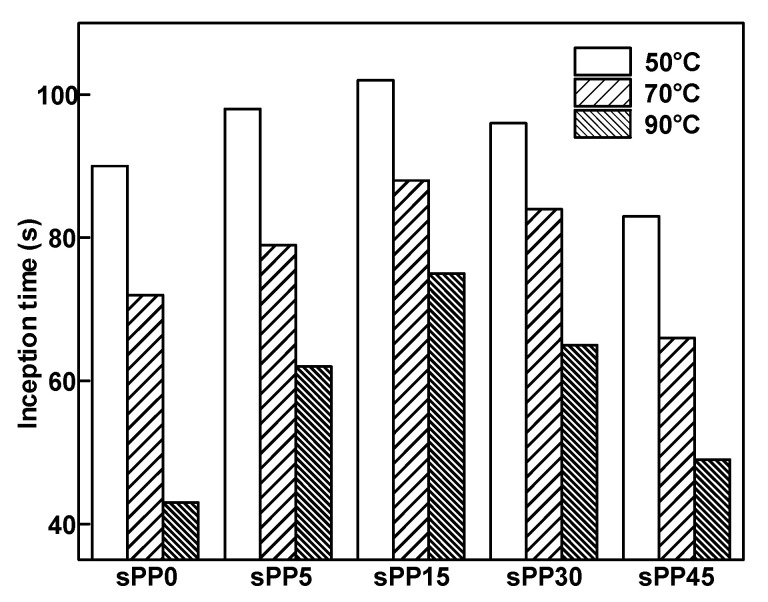
Inception time of electrical tree for isotactic polypropylene (iPP)/syndiotactic polypropylene (sPP) blends at 50, 70, and 90 °C.

Inception time is defined as the time from the beginning of voltage application to the beginning of the electrical tree. It can be observed and recorded when the length of the electrical tree is 10 µm. The inception time of the electrical tree for iPP/sPP blend insulation is shown in Figure 3. For the samples without addition of sPP, the mean inception time of electrical tree is 90 s under the ambient temperature of 50 °C. With the incorporation of sPP increasing from 0 wt% to 15 wt%, the inception time rises by 8.2%. Further improvement of sPP content decreases the inception time of the electrical tree. The inception time decreases with the increase of temperature for all the samples and decreases rapidly at high temperature. When the temperature increased from 50 °C to 90 °C, the inception time of the electrical tree in sPP0 decreased by 85%. Meanwhile, the inception time of the electrical tree in sPP15 decreased by 41%, which is rapidly less than that of sPP0.

At the temperature of 70 °C, the initiation time of sPP15 is 46% higher than that of sPP0. When the temperature rises to 90 °C, the initiation time of sPP15 is 403% higher than that of sPP0, indicating that the electrical tree resistance of pure iPP deteriorates rapidly at high temperature. The result indicates that comprising 15 wt% sPP in an iPP matrix restrains the inception of electrical tree effectively.

### 3.2. Effect of sPP Content on Electrical Tree Morphologies

The structure of the electrical tree depicts the trend of growth and propagation of the electrical tree, and the research of blending content on the morphology of the electrical tree is helpful to analyze the mechanism of blending concentration and temperature on the propagation of the electrical tree. The morphology characteristics of the electrical tree at different blending concentrations and temperatures are shown in Table 2. The brightness and contrast are adjusted to make the electrical tree clearer and easier to identify. At the temperature of 50 °C, sPP0 showed a branch like tree. With the increase of temperature, more obvious vines are distinguished at the end of branches. Compared with the branches near the tip of needle, the density of vines decreases significantly and vine tracks exhibit extremely fast growth. When the temperature further rises to 90 °C, the electrical branches are concentrated with higher growth rate. Under the temperature of 50 °C, sPP5 and sPP15 are manifested as bush like trees. With the increase of temperature, more discharge channels are generated and the structure of electrical tree gradually transformed to branch like a tree. Electrical trees of sPP30 and sPP45 indicate branch trees at 50 °C, and the density of discharge channels gradually increases with the rise of temperature.

**Figure 4:**
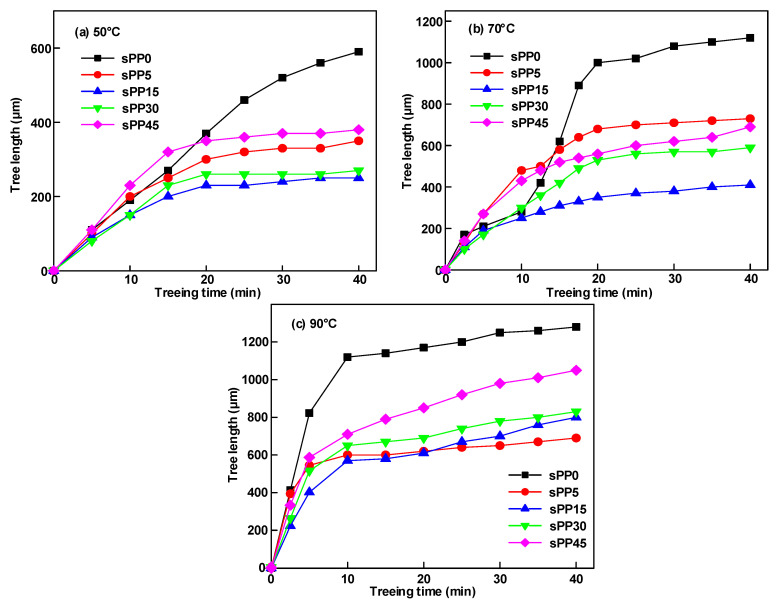
Variation of electrical tree length of different sPP contents at (**a**) 50 °C, (**b**) 70 °C, and (**c**) 90 °C under AC voltage of 6 kV.

The electrical tree length is an essential index to characterize the degradation of the electrical tree, which is closely associated with the electrical tree structure. Figure 4 shows the relationship between the electrical tree length and the experimental time under different temperatures. It can be seen from the figure that the morphology of the electrical tree displays obvious influences on the growth speed of tree channels. 

Under the temperature of 50 °C and 70 °C, the morphology of the electrical tree for sPP0 terms is a branch or branch vine mixed electrical tree. The growth rate of sPP0 suddenly increases after 15 min, reflecting the accelerated effect of the extension of the vine on the length of the electrical tree. At 90 °C, sPP0 terms branch tree with the tree length increased by 17% compared with 70 °C with the treeing time of 40 min. With the increase of the experimental temperature from 50 °C to 90 °C, the electrical tree morphology of sPP5 and sPP15 varies from bush to branch, and the electrical tree length rises enormously. Compared with sPP15, the tip dendrite of sPP5 tends to grow towards both sides at 90 °C, resulting in a relatively low growth length in longitudinal direction. sPP30 and sPP45 display a trend of transition from branch to bush with the increase of temperature, and the growth speed of the electrical tree rises.

**Figure 5:**
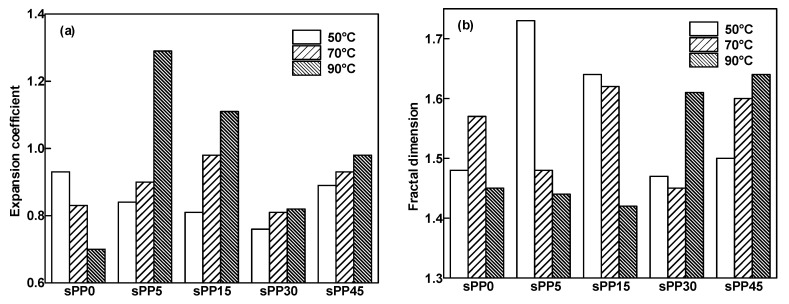
Expansion coefficient and fractal dimension of electrical tree in iPP/sPP blends at 50, 70, and 90 °C under AC voltage of 6 kV for 40 min. (**a**) Expansion coefficient, (**b**) Fractal dimension.

Fractal dimension and expansion coefficient are quantitative characterizations of electrical tree morphology, which is shown in Figure 5. With the increase of temperature from 50 °C to 70 °C, the morphology of the electrical tree of sPP0 changes from branch like tree to branch–vine mixed tree. The fractal dimension of branch like tree is smaller than that of the branch–vine mixed tree. When the temperature rises further to 90 °C, the structure of the electrical tree changes into branches with decreasing fractal dimension.

However, the expansion coefficient keeps decreasing, with indicates a more obvious transverse growth trend at higher temperatures. With the increase of temperature, the fractal dimension of the electrical tree of sPP5 and sPP15 decreases and the expansion coefficient increases, with the morphology varying from bush to branch. With the increase of temperature, the fractal dimension of the electrical tree of sPP30 and sPP45 increases and the expansion coefficient increases, with the morphology varying from branch to bush. At the temperature of 50 °C, when the content of sPP is 15% or less, the electric tree appears bush like tree. Further addition of sPP makes the electric tree a tendency change into branches. When the temperature rises to 90 °C, the variation trend of the tree structure is opposite. Further addition of sPP makes the electric tree a tendency change into bushes. The different tendency of the variation of the tree morphology at high and low temperatures makes the addition of sPP indicate an inflection point in the regulation of the electrical tree morphology at different temperatures. sPP15 distinguishes an excellent performance to inhibit electrical treeing, which benefits from the ability to promote the fractal and lateral growth of branches compared with other samples at 50 and 70 °C. When the temperature rises to 90 °C, the blends with 5 wt% sPP exhibits a better inhibition of the electrical tree than that of 15 wt%.

### 3.3. Relationship between Electrical Tree Morphologies and Propagation

**Figure 6:**
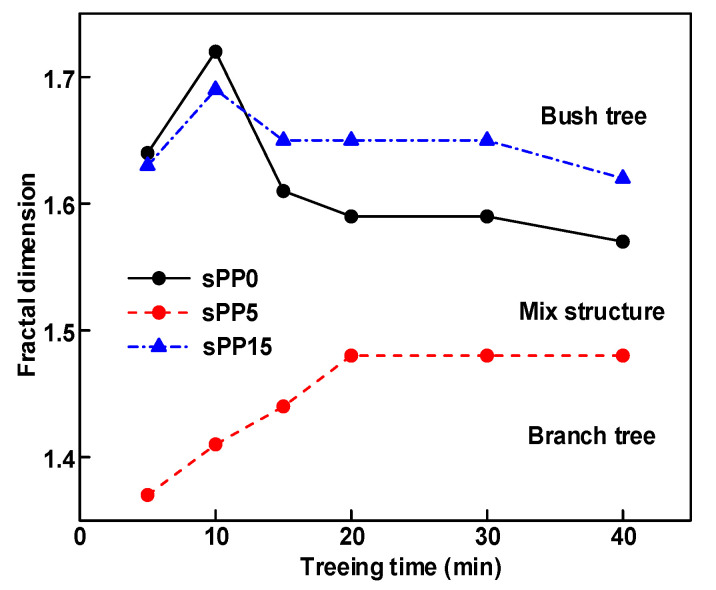
Time-varying fractal dimension of sPP0, sPP5, and sPP15 at 70 °C under AC voltage of 6 kV.

The structure description not only depends on the final state of electrical tree, but also varies and develops with the electrical tree propagation. Table 3 shows the growth process of sPP0, sPP5, and sPP15 at 70 °C, and time-varying fractal dimension of three samples is available in Figure 6. The electrical tree of sPP0 starts as a bush like tree with high fractal dimension. With 15 min of treeing time, small vines extend out of branches and grow fast with the decreasing of fractal dimension. The electrical tree of sPP5 begins to be branch like tree with a promising trend of mixed tree of branch and vine. However, the protruding vines fail to continue to grow. More dense branches are formed beside the existing branches. A dense branch like tree is formed with the fractal dimension gradually increased from 1.4 to 1.5. The red arrows point out the extending of electrical tree branches, which leads to the rapid change of fractal dimension. sPP15 presents dense bush tree from the beginning to the end, and stretching out small twigs with treeing time of 60 min, which is longer than any other sample. Dense discharge channels with less stretching branches of sPP15 inhibit the propagation of electrical tree.

The relationship between the electrical tree length and the experimental time at 70 °C can be referred to in Figure 4. It can be seen from the figure that the morphology of the electrical tree displays obvious influences on the growth speed of tree channels. The morphology of the electrical tree for sPP0 terms a branch–vine mixed electrical tree. The growth rate of sPP0 suddenly increases after 10 min, reflecting the accelerated effect of the extension of vine on the length of electrical tree. It tends to stagnate after 20 min. The extension of vine of the electrical tree of sPP5 occurs earlier than sPP0, which exhibits longer electrical tree length in the first 15 min. The electrical tree of sPP15 keeps a high fractal dimension during the experiment, which avoids the rapid change of the electrical tree length.

### 3.4. Effect of sPP Content on AC Breakdown Strength

**Figure 7:**
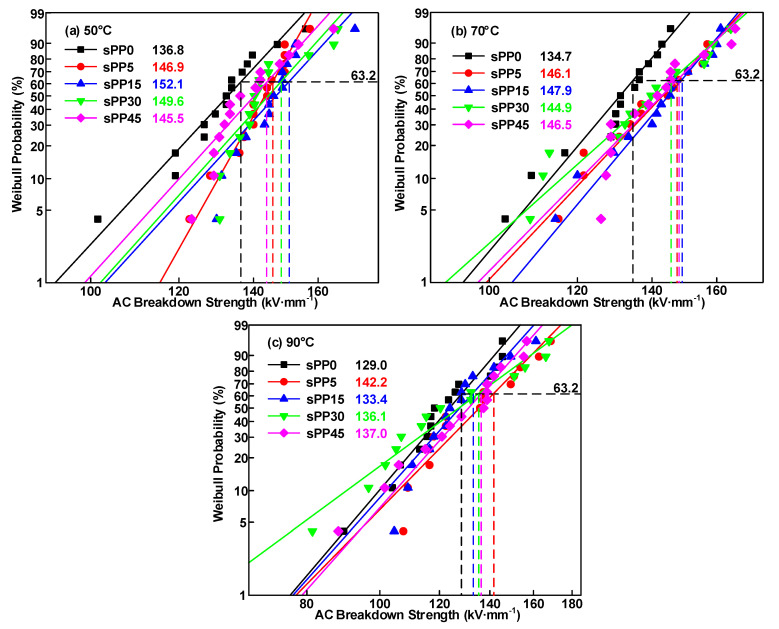
Weibull distribution of AC breakdown strength in iPP/sPP blends. (**a**) 50 °C, (**b**) 70 °C, (**c**) 90 °C.

AC breakdown field strength is a significant method to evaluate the AC withstand ability of insulating materials. Weibull distribution of AC breakdown strength of iPP/sPP blended samples is revealed in Figure 7. All the experiments maintain the same rate of voltage rise, which is 1 kV/s. It can be observed that the AC breakdown strength of the three samples shows a downward trend with the temperature rising from 50 to 90 °C, in which the breakdown field strength of PP-neat decreases from 136.8 to 129.0 kV/mm, the data of sPP5 decreases from 146.9 to 142.2 kV/mm, the data of sPP15 decreases from 152.1 to 133.4 kV/mm, the data of sPP30 decreases from 149.6 to 136.1 kV/mm and for sPP45, and the breakdown strength decreases from 145.5 to 137.0 kV/mm.

With the rising of the experimental temperature, the decrease rate of breakdown field strength is different. When the temperature increases from 50 °C to 70 °C, the breakdown field strength of the polymer decreases only within 5%, which shows the temperature-insensitive characteristic of polypropylene under AC voltage. When the content of sPP blends increases from 0 to 15 wt%, the breakdown field strength is enhanced with the rise of the sPP content. With the content of sPP blends further increasing to 45 wt%, the breakdown field strength declines. When the temperature rises to 90 °C, the breakdown field strength decreases by about 10%, and the shape parameter of Weibull distribution decreases, which indicates that the breakdown field strength of blend insulation decreases greatly at high temperature, with a more decentralized distribution.

## 4. Discussion

### 4.1. Effects of sPP Content on Crystallization

**Figure 8:**
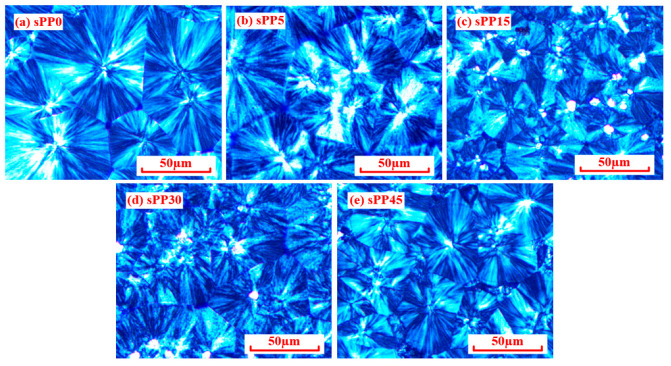
Crystal morphology of iPP/sPP blends. (**a**) sPP0, (**b**) sPP5, (**c**) sPP15, (**d**) sPP30, (**e**) sPP45.

**Figure 9:**
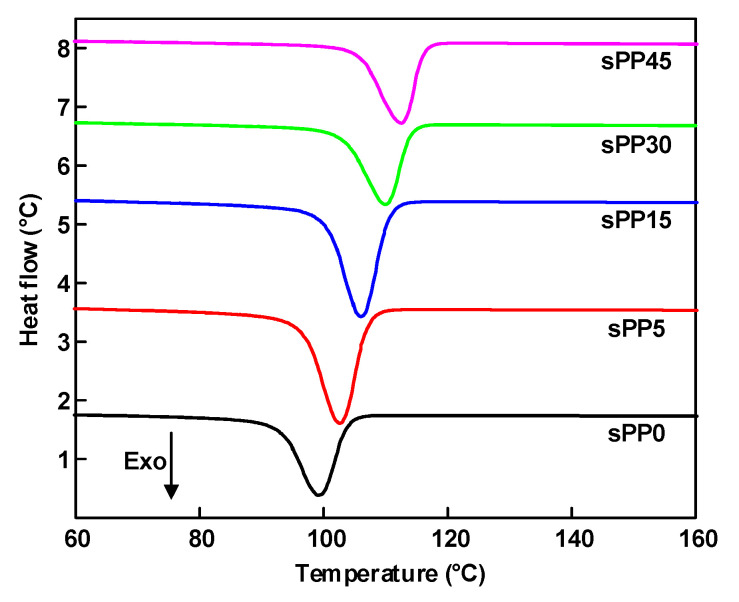
Differential scanning calorimetry (DSC) curves of iPP/sPP blends.

Polypropylene based insulating materials possess a large number of localized states distributed in amorphous regions and interface between crystalline and amorphous regions, which are accessible capturing states for charge carriers [23]. It is of great significance to investigate crystallization characteristics in order to reveal the trap mechanism. The aggregative state of blended insulation observed by polarizing optical microscope (POM) (59XF, Shanghai Optical Instrument Factory No.1) is shown in Figure 8. With the mass fraction of sPP increasing from 0 to 15 wt%, the volume of spherulites atrophies gradually with uniform distribution. With the sPP content increasing continuously, the volume of spherulites upsurges with sparse distribution. The crystallization characteristics of iPP/sPP blends were characterized by DSC test (Mettler Toledo DSC1 STARe system). Samples to be tested first rose from room temperature to 200 °C and then cooled to room temperature to eliminate the heat history. During the experiment, the temperature of samples increased from 20 °C to 200 °C at 10 K/min, and then cooled to room temperature at the same rate. Melting and crystallization curves were recorded for analysis. The crystallization curve of blends tested by DSC is illustrated in Figure 9. It can be concluded that the crystallinity of sPP0, sPP5, sPP15, sPP30, and sPP45 is 36.3%, 42.5%, 46.4%, 31.1%, and 29.0% respectively, indicating that a small amount of sPP can improve the crystallinity of the polymer.

**Figure 10:**
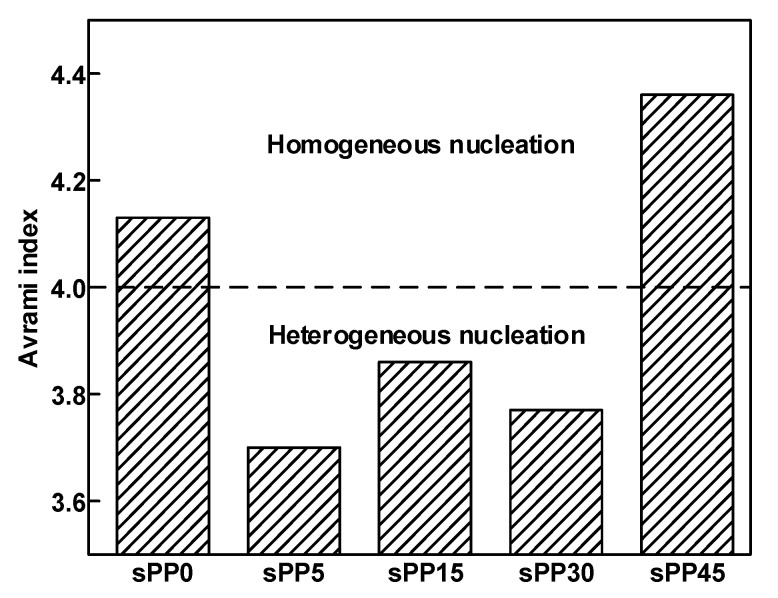
Avrami index of iPP/sPP blends.

The non-isothermal crystallization process of polymer can be roughly divided into two stages: primary crystallization and secondary crystallization. Primary crystallization is a process in which the crystal nuclei grow gradually until they collide with each other. The growth dimension of polymer crystal in primary crystallization is an important index to understand the crystallization dimension in polymer, in order to investigate the effect of crystallization on electrical properties. The values of Avrami exponent can be calculated from the fitting of Jeziorny’s improved Avrami equation in primary crystallization, which is shown as [24]:

(3) $$\log[-\ln(1-X(t))]=n\log t+\log Z_{t}$$

where *X*(*t*) represents the relative crystallinity, a function varying with crystallization time *t*, *n* is Avrami index, which is related to nucleation mechanism and growth mode, and is equal to the sum of space dimension of growth and time dimension of nucleation process. *Z_t_* represents non-isothermal crystallization rate constant. The values obtained for sPP0, sPP5, sPP15, sPP30, and sPP45 are 4.13, 3.70, 3.86, 3.77, and 4.36, respectively, as shown in Figure 10. Avrami index is generally considered as consisting of two parts: nucleation contribution and growth contribution. Considering that the crystal growth is identical, the difference of Avrami index is mainly attributed essentially to the difference in nucleation process. Therefore, with the increase of sPP content, the crystallization gradually transforms from homogeneous nucleation to heterogeneous nucleation. When the content of sPP reaches 45 wt%, the heterogeneous nucleation effect weakens.

**Figure 11:**
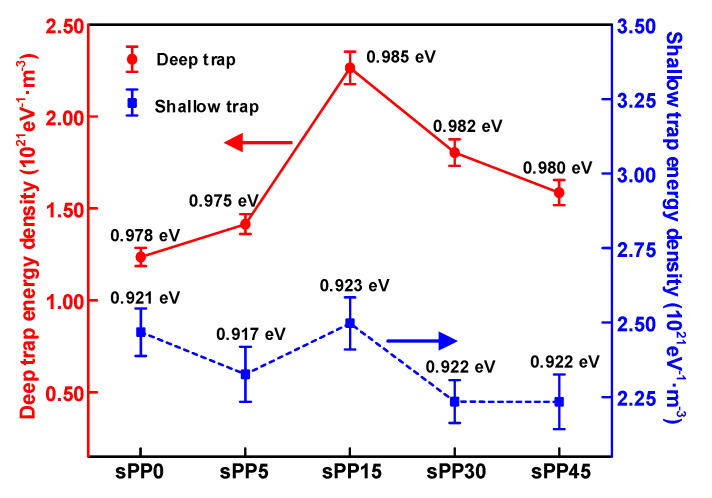
Trap level characteristics of iPP/sPP blends.

The change of crystal morphology affects localized states distributed in amorphous regions and interface between crystalline and amorphous regions, which are accessible capturing states for charge carriers. Figure 11 represents the trap energy level distribution characteristics based on IDC. It can be seen from the figure that the addition of sPP makes no difference to the trap energy level of the blend insulation. The shallow trap energy level measured by IDC remains 0.92 eV while the deep trap energy level is around 0.98 eV. The density of shallow trap is higher than deep trap. With the upsurge of sPP blending concentration, the density of shallow and deep traps of polymer insulation ascend. When the content of sPP is more than 15%, the density of shallow and deep traps declines with the rise of the blending concentration.

### 4.2. Effects of Crystallization on Electrical Tree Growth under AC Stress

The addition of sPP effectively improves the crystallization of the iPP matrix. The increase of crystallinity and heterogeneous nucleation facilitate the distribution of the interface between crystalline and amorphous regions, which limits the free transport of charge [25]. The incorporation of sPP with 15% content displays excellent crystallization with heterogeneous nucleation, thus inhibits the generation of the electrical tree.

The distortion of the electric field is the primitive driving force of electric tree propagation. The electric field varies periodically with time under AC voltage, accompanied by the electric field of space charge. At the same time, space charge will also migrate under the action of the electric field, which will conversely affect the distribution of the electric field [26]. The analysis of the variation of the electric field under AC voltage is a necessary process in the investigation of electrical tree growth.

**Figure 12:**
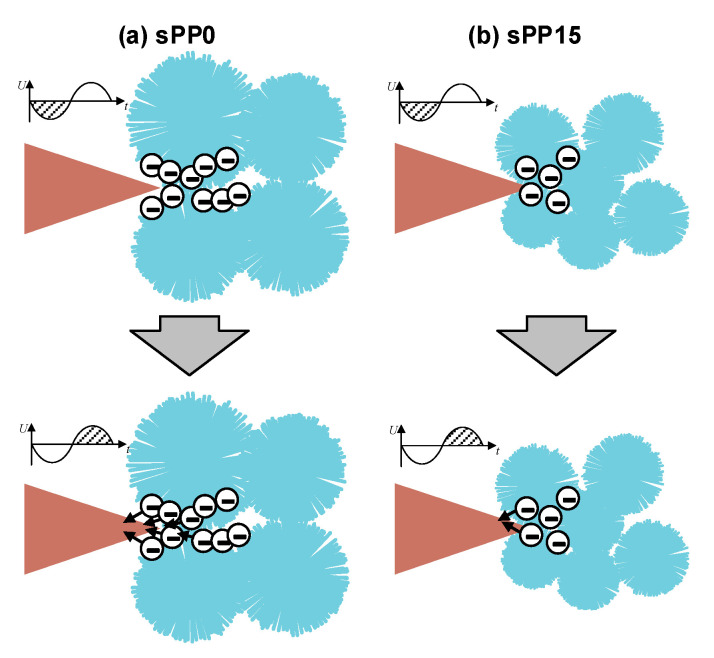
Schematic diagram of charge accumulation under AC voltage. (**a**) sPP0, (**b**) sPP15.

As shown in Figure 12, in the negative half cycle of AC voltage, electronics are injected into the polymer promoted by the electric field and trapped by traps, forming a space charge limiting electric field to inhibit the further injection of charges. When the voltage transits to the next half cycle, the injected charge will be drawn out with charge recombination. The released energy can produce energetic electrons through the Auger effect, which may destroy the molecular chain [27,28]. The residual space charges form an electric field shielding for the injected charge in the next negative half cycle.

The charge injection and extraction processes are iteratively continued, resulting in continuous energy release to promote the growth of electrical tree. The incorporation of sPP in 15 wt% introduces more deep traps into the polymer, resulting in hard extraction in reverse electric field. In the next half cycle, a stronger shielding effect is formed, which inhibits the further charge injection. Intensified density of deep traps reduces kinetic energies of electrons accelerated in free volumes [29,30,31], thus inhibits the further development of the electrical tree.

## 5. Conclusions

In this paper, the effects of sPP content of blended insulation on the growth of electrical tree and AC breakdown strength are investigated. The crystal morphology and the trap energy level characteristics of blended samples are analyzed, and a discussion based on the electric field affected by the aggregation structure has been provided to help in understanding the mechanism of microscopic crystallization on macro degradation. The conclusions are as follows:

(1) With the incorporation of sPP increasing to 15 wt%, the inception time of electrical tree increases by 8.2%. The addition of sPP by 15% distinguishes an excellent performance in inhibiting electrical treeing, which benefits from the ability to promote the fractal dimension and lateral growth of branches at 50 and 70 °C. When the temperature rises to 90 °C, the blends with 5 wt% sPP exhibits a better inhibition of the electrical tree than that of 15 wt%.
(2) With the rise of the sPP content from 0 to 15 wt%, the incorporation of sPP improves the crystallinity of polypropylene matrix and promotes heterogeneous nucleation of crystallization. Increase of crystallinity and heterogeneous nucleation introduce a large number of interfaces of crystal region and amorphous region.
(3) The introduced interface regions contain deep traps and form a space charge electric field, restraining the further injection of charges, thus inhibiting the propagation of the electrical tree. It is concluded that a 15 wt% addition of sPP exhibits a great potential application in AC cable insulation.

## Figures and Tables

**Table 1:** Catalogue of polypropylene (PP) blend samples investigated in this work.

Designation	iPP Content	sPP Content
sPP0	100 wt%	0 wt%
sPP5	95 wt%	5 wt%
sPP15	85 wt%	15 wt%
sPP30	70 wt%	30 wt%
sPP45	55 wt%	45 wt%

**Table 2:** Electrical tree morphology of iPP/sPP blends at 50, 70, and 90 °C under AC voltage of 6 kV for 40 min.

Temperature	sPP0	sPP5	sPP15	sPP30	sPP45
50 °C	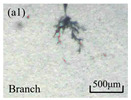	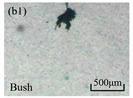	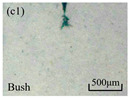	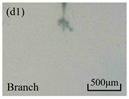	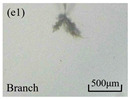
70 °C	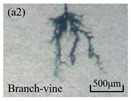	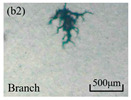	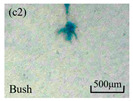	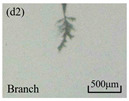	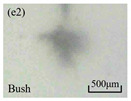
90 °C	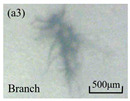	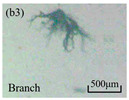	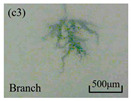	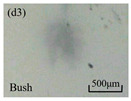	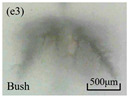

**Table 3:** Electrical tree morphology of iPP/sPP blends at 70 °C under AC voltage of 6 kV for 40 min.

Specimens	5 min	10 min	15 min	20 min	40 min
sPP0	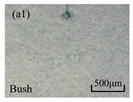	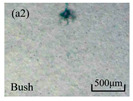	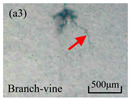	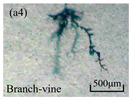	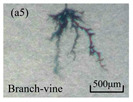
sPP5	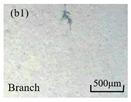	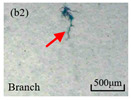	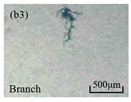	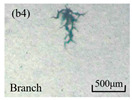	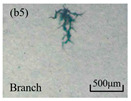
sPP15